# A Maternal Two-meal Feeding Sequence with Varying Crude Protein Affects Milk Lipid Profile in A Sow-Piglet Model

**DOI:** 10.1038/s41598-017-14188-0

**Published:** 2017-10-23

**Authors:** Xin Wu, Chunyan Xie, Xiaoyun Guo, Cimin Long, Tianyong Zhang, Tianzeng Gao, Yulong Yin

**Affiliations:** 1 Key Laboratory for Agro-Ecological Processes in Subtropical Region, Hunan Research Center of Livestock & Poultry Sciences, South-Central Experimental Station of Animal Nutrition and Feed Science in Ministry of Agriculture, Institute of Subtropical Agriculture, the Chinese Academy of Sciences, Changsha, Hunan 410125 China; 2 College of bioscience and biotechnology, Hunan Agricultural University, Changsha, Hunan 410128 China; 3 Henan Guang’an Biology Technology Co. Ltd., Zhengzhou, 450001 China

## Abstract

The effects of a two-meal feeding sequence on production performance and milk lipid profile were investigated. Sixty pregnant sows (d 85 of gestation) were assigned to 3 groups: 2 C group (fed a control crude protein [CP] diet at 0600 and 1500 daily), LH group (fed a low CP diet and a high CP diet at 0600 and 1500), or HL group (fed a high CP diet and a low CP diet at 0600 and 1500). Reproductive performance of sows, and lipid profiles of plasma and milk were measured. Results showed that the HL feeding sequence dramatically increased average piglet weight/litter, average daily gain of piglet/litter, and milk production of sows. LH feeding sequence increased milk fat proportion, and HL feeding sequence significantly increased the proportion of milk MUFA on d 14 and 21 of lactation. Interestingly, the HL feeding sequence also reduced the ratio of C18:1_***cis***_/C18:1_***trans***_ in milk, which may account for the greater milk production of sows and growth performance of piglets during lactation. These findings indicated that both the maternal two-meal feeding sequences with varying crude protein improved milk production and milk lipid profiles of sows, which might contribute to improving growth performance of piglets.

## Introduction

Feeding sows during the transition from late gestation to lactation is important for the production of colostrum and milk, which is related to the lactation performance of sows and growth rates of piglets^1^. Milk is the sole source of nutrients for the neonates of most mammalian species^2^. As the number of nursing piglets per sows has increased in recent years, lactating sows have not provided enough milk to satisfy the needs of rapidly growing piglets^3^. Consequently, improving milk production and quality are important for the productivity performance of sows. Current feeding systems related to phase-feeding programs and feeds are usually formulated to optimise the performance of the total pig population, despite the fact that in such evaluations, most of the pigs receive more nutrients than they actually need for optimal growth^4^. Recent studies showed that nutritional outcomes were affected by the timing of food intake, even when the same type of food and the same amount of calories were consumed, as circadian clocks and energy metabolism interacted with each other^5,6^. Therefore, optimised feeding systems linking dietary formulation and nutritional management techniques to circadian clocks would significantly improve animal husbandry.

Previous studies have reported that feeding time affects body weight and the risk of obesity in humans^7,8^. Our previous study showed that a daily meal sequence with different dietary crude protein (CP) affected the metabolism and growth performance in growing pigs^9^, and improved muscle quality characteristics^10^. As one of the most important parts of milk, fat or fatty acid is important for offspring growth^11^. However, little is known about how a maternal two-meal feeding sequence affects the milk composition of sows and growth rates of piglets.

In this study, swine models were chosen for their physiological and gastrointestinal similarities to humans. Moreover, sows and neonatal piglets are more similar in size to human mothers and infants than other animal models, such as mice^12^. Our previous study demonstrated that feeding a high protein meal in the morning and a gradually reduced CP content in meals during the day affected lipid metabolism in barrows^10^. In addition, we found that long chain fatty acid contents in plasma and liver both exhibited diurnal rhythms in growing pigs^13^. Therefore, this study investigated the effects of different feeding sequences on milk production, the lipid profiles of sows, and growth performance of their offspring. We hypothesised that the maternal HL two-meal sequence with varying CP would increase the growth performance of neonatal piglets through the mother-to-newborn transfer of milk.

## Results

### Reproductive performance of sows

Descriptive data on the reproductive performance of sows are presented in Table 1. On average, piglet weights from the 2 C group at d 14 (*P* = 0.04) and d 21 (*P* = 0.024) were lower than those from sows in the LH or HL groups; however, there were no significant differences in piglet birth weights among the three groups (*P* = 0.15). Moreover, both the HL and LH feeding sequences significantly increased the average daily gain of piglets/litter during d 0–21 of lactation (*P* = 0.034), and by 14.22% and 10.22% during d 14–21 of lactation (*P* = 0.17), respectively. Additionally, the average daily gain of piglets/litter also showed an increasing trend in the HL group compared with the LH and 2 C groups (*P* = 0.054). However, no significant difference was observed between groups in the total number of newborn piglets/litter, stillbirth number/litter, or litter size at weaning (*P* > 0.10).

**Table 1:** Reproductive performance of sows according to feeding sequence^1,2^.

Item	Feeding sequence			SEM	*P*-value
	2 C	LH	HL		
Total number of newborn piglets/litter	13.21	12.58	12.67	0.376	0.77
Stillbirth number/litter	1.100	1.105	0.952	0.135	0.87
Litter size at weaning	11.47	11.16	11.05	0.340	0.87
Average piglet’s weight (kg)/litter					
Birth weight	1.358	1.454	1.447	0.022	0.15
On d 14	4.031^b^	4.157^ab^	4.575^a^	0.094	0.04
On d 21	5.604^b^	5.894^ab^	6.376^a^	0.121	0.024
Average daily gain of piglets (kg)/litter					
d 0–14	0.191^b^	0.194^b^	0.223^a^	0.006	0.054
d 14–21	0.225	0.248	0.257	0.007	0.17
d 0–21	0.202^b^	0.212^ab^	0.235^a^	0.005	0.034

^a–b^Values with different letters within the same row are different (*P* < 0.05).

^1^2C = fed control diet at 0600 h and 1500 h a day, respectively, LH = fed low protein diet at 0600 h and high protein diet at 1500 h a day, HL = fed high protein diet at 0600 h and low protein diet at 1500 h a day, respectively.

^2^n = 20.

### Milk production

**Figure 1:**
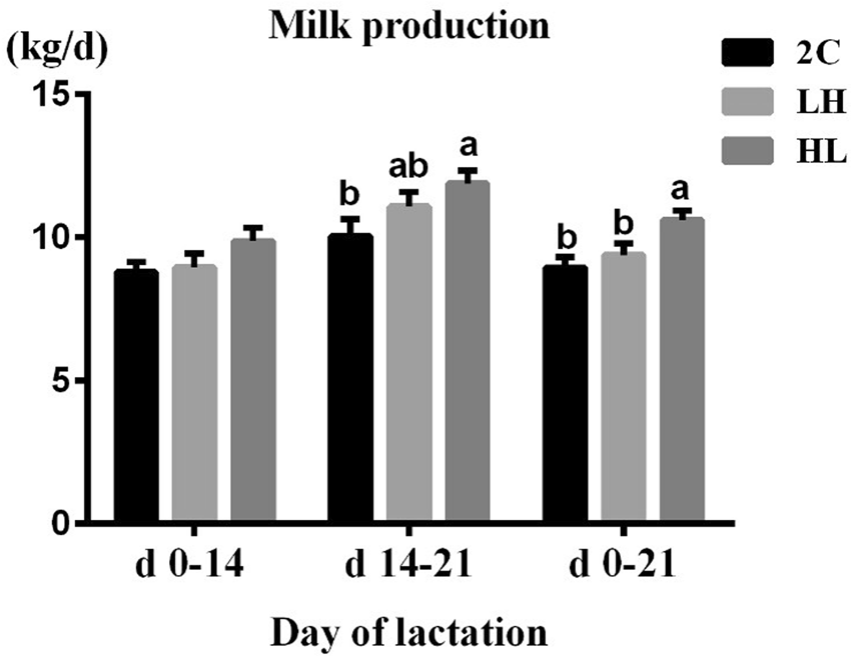
Milk production of sows according to 2 C, LH and HL feeding sequences. Values are means + SEM, and a, b were used to indicate a statistically significant difference (*P* < 0.05, one-way ANOVA method).

Based on the growth rates of piglets/litter (per sow), we found that the HL feeding sequence increased milk production per sow during d 14–21 (*P* = 0.049) and d 0–21 (*P* = 0.007) of lactation, whereas no significant difference was observed among the three groups in milk production during d 0–14 of lactation (*P* = 0.16) (Fig. 1).

### Plasma lipid profiles of sows

As shown in Table 2, the plasma H-DLC/total cholesterol (CHO) ratio in sows in the HL group was greater than that in sows in the LH and 2 C groups (*P* < 0.05). However, other plasma lipid parameters were not significantly affected by feeding sequences. After 14 days of lactation, plasma H-DLC (*P* = 0.014), L-DLC (*P* = 0.038), and total CHO (*P* = 0.031) declined in the HL group compared with those in the 2 C group. Additionally, the ratio of both H-DLC/L-DLC (*P* = 0.012) and H-DLC/total CHO (*P* = 0.003) in the LH group declined, but the HL group was not affected compared with the 2 C group (*P* > 0.10). Plasma triglyceride in sows was not significantly affected by feeding sequences during late gestation or lactation (*P* > 0.10).

**Table 2:** Plasma lipid profiles in sows according to feeding sequence^1,2^.

Item	Feeding sequence			SEM	*P*-value
	2 C	LH	HL		
Plasma of sows during farrowing					
H-DLC (mmol/l)	1.026	1.078	0.990	0.052	0.802
L-DLC (mmol/l)	0.611	0.746	0.590	0.037	0.169
Total CHO (mmol/l)	1.578	1.773	1.465	0.070	0.200
Triglyceride (mmol/l)	0.256	0.300	0.243	0.015	0.300
H-DLC/L-DLC	1.685	1.463	1.770	0.077	0.256
H-DLC/total CHO	0.641^ab^	0.607^b^	0.710^a^	0.017	0.036
L-DLC/total CHO	0.391	0.417	0.399	0.011	0.615
Plasma of sows on d 14 of lactation					
H-DLC (mmol/l)	1.168^a^	0.866^b^	0.881^b^	0.052	0.014
L-DLC (mmol/l)	1.114^a^	1.068^a^	0.860^b^	0.045	0.038
Total CHO (mmol/l)	2.293^a^	1.976^ab^	1.695^b^	0.101	0.031
Triglyceride (mmol/l)	0.178	0.224	0.220	0.014	0.299
H-DLC/L-DLC	1.053^a^	0.810^b^	1.014^a^	0.036	0.012
H-DLC/total CHO	0.509^a^	0.438^b^	0.515^a^	0.011	0.003
L-DLC/total CHO	0.491	0.542	0.508	0.010	0.135

^a–b^Values with different letters within the same row are different (*P* < 0.05).

^1^2C = fed control diet at 0600 h and 1500 h a day, respectively, LH = fed low protein diet at 0600 h and high protein diet at 1500 h a day, HL = fed high protein diet at 0600 h and low protein diet at 1500 h a day, respectively.

^2^n = 10.

### Proportion of fat and fatty acids profiles in milk

The proportions of fat and fatty acids profiles in milk on d 7 are listed in Table 3. Total proportion of MUFA (*P* = 0.003), C22:6 (DHA) (*P* = 0.011), C22:2 (*P* < 0.0001), C20:3 (*P* = 0.034), C20:2 (*P* = 0.025), and C18:1*trans* (*P* < 0.0001) in milk increased, but C16:0 (*P* = 0.025), C17:0 (*P* = 0.005), C18:1*cis* (*P* < 0.001), C18:2*trans* (*P* = 0.004), total SFA (*P* = 0.021), and C18:1_*cis*_/C18:1_*trans*_ ratio (*P* < 0.0001) in milk decreased in the HL feeding group compared with those in the 2 C and LH groups. The LH feeding sequence significantly reduced the proportions of C18:3 (*P* < 0.001) and n-3 (*P* = 0.004), and increased the ratio of n-6/n-3 (*P* < 0.001). However, the proportion of fat in milk was not affected by the different feeding sequences.

**Table 3:** Medium-long-chain fatty acids profile and fat proportion in milk according to feeding sequence on d 7^1,2,3^.

Item	Feeding sequence			SEM	*P*-value
	2 C	LH	HL		
*Fat and fatty acid value (%)*					
C10:0	0.342^a^	0.323^ab^	0.293^b^	0.010	0.103
C12:0	0.415	0.411	0.402	0.010	0.814
C14:0	3.954	3.882	3.897	0.070	0.932
C14:1	0.276	0.283	0.272	0.010	0.910
C15:0	0.113	0.126	0.122	0.005	0.525
C15:1	0.314^a^	0.297^a^	0.115^b^	0.040	0.044
C16:0	32.33^a^	31.67^ab^	29.94^b^	0.370	0.025
C16:1	11.09	11.03	10.33	0.250	0.385
C17:0	0.499^a^	0.512^a^	0.282^b^	0.030	0.005
C17:1	0.108^b^	0.153^a^	0.102^b^	0.010	0.045
C18:0	3.998	3.937	3.546	0.130	0.218
C18:1 *cis*	5.340^a^	4.923^a^	3.520^b^	0.220	<0.001
C18:1 *trans*	18.99^b^	21.11^b^	24.69^a^	0.550	<0.0001
C18:2 *cis*, n − 6	18.77	17.13	18.70	0.440	0.210
C18:2 *trans*, n − 6	0.148^a^	0.148^a^	0.085^b^	0.010	0.004
C18:3, n − 3	1.338^a^	1.089^c^	1.222^b^	0.030	<0.001
C18:3, n − 6	0.135	0.153	0.143	0.010	0.753
C20:0	0.222	1.255	0.158	0.320	0.292
C20:1	0.086^a^	0.056^ab^	0.051^b^	0.007	0.091
C20:2	0.218^b^	0.233^b^	0.326^a^	0.020	0.025
C20:3, n − 3	0.037	0.034	0.042	0.001	0.119
C20:3, n − 6	0.082^b^	0.089^ab^	0.101^a^	0.003	0.034
C20:4, n − 6	0.526	0.487	0.557	0.015	0.122
C21:0	0.051	0.063	0.060	0.007	0.782
C22:0	0.035	0.046	0.044	0.005	0.640
C22:2	0.064^b^	0.070^b^	0.093^a^	0.003	<0.0001
C22:6, n − 3	0.268^b^	0.283^b^	0.339^a^	0.010	0.011
MUFA	36.27^b^	37.92^a^	39.14^a^	0.343	0.003
PUFA	21.59	20.71	21.61	0.332	0.440
SFA	42.26^a^	42.53^a^	39.16^b^	0.583	0.021
n − 6	19.66	18.99	19.59	0.300	0.610
n − 3	1.643^a^	1.406^b^	1.604^a^	0.033	0.004
Fat	7.430	7.713	7.474	0.174	0.790
*The ratio of fatty acids*					
C18:1*cis*/C18:1*trans*	0.293^a^	0.239^a^	0.142^b^	0.014	<0.0001
n − 6/n−3	12.12^b^	13.62^a^	12.27^b^	0.190	<0.001

^a^ − ^c^Values with different letters within the same row are different (*P* < 0.05).

^1^2 C = fed control diet at 0600 h and 1500 h a day, respectively, LH = fed low protein diet at 0600 h and high protein diet at 1500 h a day, HL = fed high protein diet at 0600 h and low protein diet at 1500 h a day, respectively.

^2^The percentage values are proportions of each individual fatty acid relative to the total detectable lipid in the sample.

^3^n = 10.

Similar trends in MUFA (*P* < 0.0001), C16:0 (*P* < 0.001), C17:0 (*P* = 0.008), C20:3 (*P* < 0.001), C20:2 (*P* < 0.0001), C18:1*cis* (*P* < 0.001), C18:1*trans* (*P* < 0.001), C18:1*cis*/C18:1*trans* (*P* < 0.0001), and total SFA (*P* = 0.002) were observed on day 14 (Table 4 **)**. Additionally, both HL and LH feeding sequences increased the proportions of C18:3, n-6 (*P* < 0.001), C11:0 (*P* < 0.0001), and C17:1 (*P* < 0.001) in milk compared with those in 2 C, whereas the proportion of C20:0 (*P* = 0.022) in the LH group was greater than that in the other two groups. Compared with the 2 C group, the proportion of n-6 in the HL and LH groups presented an increasing trend (0.05 < *P* < 0.10), n-3 was decreased (*P* < 0.0001), and the n-6/n-3 ratio was significantly increased (*P* = 0.004). Notably, the proportion of fat was highest in the LH group (*P* = 0.0005).

**Table 4:** Medium-long-chain fatty acids profile and fat proportion in milk according to feeding sequence on d 14^1–3^.

Item	Feeding sequence			SEM	*P*-value
	2 C	LH	HL		
*Fat and fatty acid value (%)*					
C10:0	0.374	0.343	0.373	0.012	0.496
C11:0	0.049^b^	0.161^a^	0.173^a^	0.014	<0.0001
C12:0	0.470	0.464	0.464	0.007	0.939
C14:0	4.340	4.097	4.091	0.057	0.150
C14:1	0.346	0.327	0.362	0.012	0.486
C15:0	0.117	0.109	0.121	0.005	0.528
C16:0	33.64^a^	31.20^b^	29.96^b^	0.404	<0.001
C16:1	12.02	10.94	11.01	0.303	0.312
C17:0	0.312^a^	0.198^b^	0.142^b^	0.023	0.008
C17:1	0.102^b^	0.163^a^	0.207^a^	0.011	<0.001
C18:0	3.954	4.090	3.711	0.082	0.174
C18:1*cis*	2.937^a^	2.362^b^	2.307^b^	0.079	<0.001
C18:1*trans*	20.66^b^	23.84^a^	25.68^a^	0.552	<0.001
C18:2*cis*, n − 6	17.04^b^	18.01^a^	17.47^ab^	0.186	0.113
C18:3, n − 3	1.321	1.191	1.242	0.045	0.448
C18:3, n − 6	0.088^b^	0.114^a^	0.125^a^	0.004	<0.001
C20:0	0.105^ab^	0.120^a^	0.079^b^	0.006	0.022
C20:1	0.033^c^	0.143^b^	0.245^a^	0.016	<0.0001
C20:2	0.122^b^	0.299^a^	0.351^a^	0.022	<0.0001
C20:3, n − 6	0.069^c^	0.120^a^	0.097^b^	0.006	<0.001
C20:4, n − 6	0.392^b^	0.448^ab^	0.475^a^	0.012	0.020
C22:6, n − 3	0.340^a^	0.289^b^	0.221^c^	0.012	0.0001
MUFA	36.10^c^	37.77^b^	39.82^a^	0.367	<0.0001
PUFA	19.37^b^	20.47^a^	19.98^ab^	0.215	0.127
SFA	43.74^a^	41.39^b^	39.88^b^	0.455	0.002
n − 6	17.58^b^	18.69^a^	18.16^ab^	0.193	0.074
n − 3	1.839^a^	1.481^b^	1.464^b^	0.038	<0.0001
Fat	6.988^b^	7.976^a^	6.854^b^	0.148	0.003
*The ratio of fatty acids*					
C18:1*cis*/C18:1*trans*	0.147^a^	0.099^b^	0.090^b^	0.005	<0.0001
n − 6/n − 3	9.653^b^	13.46^a^	12.45^a^	0.497	0.004

^a–c^Values with different letters within the same row are different (*P* < 0.05).

^1^2C = fed control diet at 0600 h and 1500 h a day, respectively, LH = fed low protein diet at 0600 h and high protein diet at 1500 h a day, HL = fed high protein diet at 0600 h and low protein diet at 1500 h a day, respectively.

^2^The percentage values are proportions of each individual fatty acid relative to the total detectable lipid in the sample.

^3^n = 10.

The proportion of fat and fatty acids profiles in milk on day 21 are listed in Table 5. MUFA (*P* < 0.0001), n-3 (*P* < 0.0001), C18:1*trans* (*P* < 0.0001), the ratio of n-6/n-3 (*P* < 0.0001), and C18:1_*cis*_/C18:1_*trans*_ (0.05 < *P* < 0.10) presented similar quantities as observed on days 7 and 14. Compared with the 2 C group, the proportion of C22:6 (*P* < 0.0001), C18:3, n-3 (*P* < 0.0001), and C14:1 (*P* < 0.0001) were decreased in the HL group. However, C15:0 (*P* < 0.0001) and C20:2 (*P* = 0.023) were increased in the HL and LH groups, respectively. The proportion of fat was also increased in the LH group (*P* = 0.012) compared with that in the 2 C and HL groups. However, the proportion of n-6 in milk on d 7, 14, and 21 was not affected by different feeding sequences.

**Table 5:** Medium-long-chain fatty acids profile and fat proportion in milk according to feeding sequence on day 21^1–3^.

Item	Feeding sequence			SEM	*P*-value
	2 C	LH	HL		
Fat and fatty acid value (%)					
C10:0	0.365^a^	0.342^ab^	0.313^b^	0.009	0.081
C12:0	0.480	0.479	0.483	0.007	0.968
C14:0	4.183	4.126	4.234	0.061	0.780
C14:1	0.359^a^	0.293^a^	0.135^b^	0.020	<0.0001
C15:0	0.111^b^	0.113^b^	0.294^a^	0.018	<0.0001
C16:0	32.75	31.68	31.24	0.414	0.334
C16:1	12.12	11.56	11.34	0.271	0.495
C17:1	0.101	0.108	0.117	0.013	0.876
C18:0	1.475	0.925	1.548	0.018	0.338
C18:1*cis*	2.892^b^	3.512^a^	3.185^ab^	0.414	0.005
C18:1*trans*	21.96^b^	26.95^a^	27.44^a^	0.271	<0.0001
C18:2*cis*, n − 6	17.68	17.90	18.14	0.013	0.757
C18:3, n − 3	1.664^a^	1.343^b^	1.437^b^	0.032	<0.0001
C20:2	0.201^b^	0.285^a^	0.210^b^	0.017	0.023
C22:6, n − 3	0.232^a^	0.148^b^	0.161^b^	0.007	<0.0001
MUFA	38.45^b^	42.42^a^	42.21^a^	0.374	<0.0001
PUFA	20.27	19.67	19.95	0.223	0.590
SFA	40.09	37.76	38.19	0.505	0.137
n − 6	18.12	17.90	18.14	0.199	0.877
n − 3	1.941^a^	1.491^b^	1.598^b^	0.036	<0.0001
Fat	6.484^b^	7.633^a^	6.755^b^	0.167	0.012
The ratio of fatty acids					
C18:1*cis*/C18:1*trans*	0.131	0.132	0.117	0.003	0.091
n − 6/n − 3	9.386^c^	12.07^a^	11.37^b^	0.178	<0.0001

^a–c^Values with different letters within the same row are different (*P* < 0.05).

^1^2C = fed control diet at 0600 h and 1500 h a day, respectively, LH = fed low protein diet at 0600 h and high protein diet at 1500 h a day, HL = fed high protein diet at 0600 h and low protein diet at 1500 h a day, respectively.

^2^The percentage values are proportions of each individual fatty acid relative to the total detectable lipid in the sample.

^3^n = 10.

## Discussion

Energy metabolism and circadian clocks interaction as well as nutritional outcomes are affected by the timing of food intake, even when the same type of food and the same amount of calories are consumed^14^. A previous study reported that plasma triglycerides show diurnal variation^15^. Our results also showed that the plasma lipid profiles of sows, especially on d 14 of lactation, was influenced by HL and LH feeding patterns, whereas the plasma triglyceride levels in sows did not show a significant difference among different groups. Moreover, changes in plasma CHO levels in sows suggested that a daily two-meal feeding sequence with different dietary CP, especially the HL feeding sequence, reduced the uptake of CHO or *de novo* synthesis of CHO in the liver. Notably, the plasma lipid profiles of sows at farrowing represented the late gestation period to some extent, and did not show significant differences between the three groups compared with those during lactation. These findings may be explained by the more vigorous lipid metabolism during lactation than gestation, but further study is needed to verify this.

A previous study reported that neither the volume of milk production nor the total milk lipid concentration was related to the variation in composition or energy content of the maternal diet^16^, but was strongly influenced by litter size, piglet weight, and suckling interval^17,18^. Milk production will increase if piglets suckle more frequently. In this study, the HL feeding sequence resulted in the maximum daily milk production/sow determined by litter size and average daily gain of piglets, although we did not evaluate the suckling intervals of piglets/litter. Growth rates of piglets were partially determined by the higher daily milk performance of sows. Normally, piglets that are heavier at birth grow faster during the suckling period because they are able to massage the teats more strongly and obtain more milk at each suckling^19^. These findings suggested that the HL feeding sequence was the most vigorous in stimulating a greater milk flow, which then helped to improve the growth rates of piglets.

Nutrients are supplied to the newborn through milk, and fat is particularly important not only for energy, but also for supplying specific fatty acids that are essential for optimal organ development^20^. Fat-free diets fed to infants increased energy requirements and reduced growth^21^, which indicated that milk fat was of great significance in the development of offspring. Some studies reported that rats and sows fed high-lipid diets during pregnancy and lactation had higher daily outputs of total lipid in milk and their offspring had higher growth rates^22,23^. It is reported higher piglet growth due to a higher milk fat content^24^. Our results indicated that the LH feeding sequence may resolve the problems associated with low milk fat content in sows, which also provides information for maternal nutrition in humans, cows, and other mammals. However, in this study, the increase in milk fat in sows from the LH group was not sufficient to account for the maximum growth rates of piglets.

Maternal nutrition has little or no effect on many nutrients in human milk, except that fatty acids show extreme sensitivity to maternal nutrition and are implicated in neurological development^25^. The utilisation of energy reserves is reflected in the content of milk fat^26^, fatty acids profile, and the mutual ratio between individual fatty acid proportions in milk^27^. Recently, dietary PUFA in feeds, specifically the n-3 and n-6 fatty acids, have been studied to improve sow and piglet performance. Moreover, it has been demonstrated that animal performance was influenced by the ratio of n-6/n-3 in pigs^28^. Conversely, it is reported that changing the n-6/n-3 fatty acid ratio in sow diets changed colostrum and milk fatty acids profiles, but had minimal impact on reproductive performance^29^. In this study, we found that both the LH and HL feeding sequences affected the proportion of n-3 fatty acids and the ratio of n-6/n-3 in milk. However, these changes did not decrease the growth performance of piglets, which was consistent with the previous study, although robust evidence supporting a direct link between the proportion of milk n-3 fatty acids, n-6/n-3 ratio, and growth performance of suckling piglets is limited.

One study showed that altering the conformation of a C18:1 double bond from cis to trans (oleic acid to elaidic acid) in *in vitro* cellular models decreased cholecystokinin secretion, a satiety hormone involved in appetite regulation^30^. In this study, the LH and HL feeding sequences significantly increased the proportion of C18:1*trans* in milk on days 7, 14, and 21 of lactation, but decreased the proportion of C18:1*cis* and the ratio of C18:1*cis*/C18:1*trans*, which is related to the appetite of suckling piglets. Again, this result indicated that milk production of sows could be increased via the two-meal feeding sequence, especially the HL sequence, to improve the appetite of suckling piglets and milk production of sows, although the latter point needs more studies of suckling behaviour to verify.

Besides C18:1 MUFA, the LH and HL feeding sequences also resulted in a greater total MUFA and lower proportion of SFA in milk. Although there is no conclusive evidence that high levels of SFA in the diet are directly linked to cardiovascular disease, it has been demonstrated that diets with high MUFA could protect against this disease^31^. Furthermore, it is found that a diet containing a high proportion of SFA reduced feed intake more than one rich in unsaturated fatty acids^32^. In agreement with a previous study, changes in SFA, C18:1 MUFA, and total MUFA, which are involved in appetite regulation, may explain the optimal milk composition in the HL group. Sow milk composition and production are important factors in determining mortality and growth rates of pre-weaning piglets^2^. In other words, our data indicated that the HL feeding sequence may improve the growth rates of piglets via altering conformation of C18:1 and SFA proportions in milk that regulate the appetites of piglets, and in turn improve the milk production of sows. Interestingly, the proportion of DHA in milk on d 7 of lactation increased in the HL group, but significantly decreased on days 14 and 21 in the LH and HL groups. The possible reasons for this variation need further study.

Milk fat comes from two sources: biosynthesis of fatty acids within the mammary glands (*de novo* synthesis) and uptake from the plasma by the mammary glands^22^, both of which are influenced by maternal nutrition^33,34^. Milk fatty acids profiles are dependent on maternal dietary lipid intake^35,36^. In contrast to a former study^20^, the daily two-meal feeding sequence with varying CP (HL or LH) affected the milk fat content and fatty acids profiles of sows despite constant dietary lipid content. These findings indicated that the growth performance of piglets could be improved without using additives in sow feeds, but by applying a maternal dynamic feeding sequence (i.e., HL or LH) compared with conventional feeding, i.e., the 2 C feeding sequence. Consequently, we suggest that the LH feeding sequence could improve the biosynthesis of fatty acids or absorption by the mammary glands in sows. However, to further understand its modulation by maternal dietary protein, further studies are needed.

A previous study demonstrated that the chemical composition of human milk is modified according to pregnancy maturation (pre- and post-partum) and milking time (night versus day time), and that these differences are adapted to the nutritional needs of the infant^37^, which was consistent with the results of milk fatty acids profiles in sows on d 7, 14 and 21 of lactation.

## Conclusions

Compared to the conventional feeding sequence (2 C), dynamic maternal nutrition of a maternal two-meal with varying CP modulated milk production and lipid profiles in milk and plasma of sows. In turn, this contributed to the greater growth performance of piglets. These findings may be useful for maximising milk production of sows without increasing feed costs, and providing information for mother-infant nutrition in humans.

## Materials and Methods

This study was conducted according to the guidelines for the treatment of animal subjects as approved by the Animal Care Committee of the Institute of Subtropical Agriculture, Chinese Academy of Science.

### Animals, experimental design, and diets

The diets for sows in the experiment included a control diet (2 C), high-protein diet (HL), and a low protein diet (LH). A total of 60 pregnant sows (Landrace × Large Yorkshire) with a similar parity (3–6) were randomly assigned to one of 3 groups (20 replicates/group): 2 C (sows were fed a basal diet with 19.68% crude protein [CP] at 0600 h and 1500 h daily), LH (sows were fed a basal diet with 18.09% CP and 21.28% CP at 0600 h and 1500 h daily, respectively), and HL (sows were fed a basal diet with 21.28% CP and 18.09% CP at 0600 h and 1500 h daily, respectively).

Gestating sows were fed with the 2 C CP diet until 4 weeks pre-farrowing (d 85 ± 2 of gestation), when they began consuming their assigned treatment diet until weaning. Sows in the HL and LH groups were fed with the same quantity of diets at 0600 h and 1500 h throughout the experiment to ensure they all consumed equal amounts of feed with the same CP content daily.

During gestation, sows were housed in 8 pens, each with 6 individuals, walk in/lock in stalls. On d 110 of gestation, 20 sows were moved from the gestation facility to a farrowing room equipped with 20 individual farrowing crates. Each crate was 183 cm wide and 244 cm long, and had an adjustable sow space and a piglet creep area. All sows were housed individually in an environmentally controlled nursery and had free access to fresh water throughout the experiment. The piglets were suckled only by the sows and received no additional feed until weaning (aged 21 d).

In this study, the nutrients in feed were considered to be adequate for sows and met the NRC-recommended requirements within the appropriate weight range (NRC, 2012). The compositions of the diets are given in Table 6.

**Table 6:** Composition of the sow diets (as-fed basis).

Ingredient (%)	Diets		
	Control	High CP	Low CP
Corn	30.0	26.5	33.5
Wheat	29.9	29.9	29.9
Wheat bran	6.0	5.0	7.0
DDGS	5.0	5.0	5.0
Soybean meal, CP 46%	19.94	22.94	16.94
Fish meal, CP 65%	2.5	3.5	1.5
Chocolate powder	2.0	2.0	2.0
Soy oil	1.0	1.5	0.5
Salt	0.3	0.3	0.3
CaCO_3_	0.82	0.82	0.82
CaHPO_4_, 23/17	0.97	0.97	0.97
Limestone	0.26	0.26	0.26
Lys, 78.8%	0.05	0.05	0.05
Choline	0.21	0.21	0.21
Flavoring agent^1^	0.05	0.05	0.05
Vitamin-mineral Premix^2^	1	1	1
**Nutrient composition**			
CP, %	19.68	21.28	18.09
ME, MJ/kg	13.50	13.65	13.35
Crude Fat	4.03	4.48	3.58
Crude fibre	3.061	3.060	3.062
Crude ash	5.38	5.58	5.19
Salt	0.34	0.35	0.33
Ca	0.79	0.82	0.75
Available P	0.46	0.49	0.43
Lys	0.97	1.09	0.85
Met	0.34	0.37	0.32
Cys	0.31	0.33	0.29
Thr	0.694	0.759	0.628
Trp	0.211	0.231	0.191

^1^Flavoring agent means flavor and sweetener.

^2^Premix provided the following per kg of diet: Fe (FeSO4·H2O), 80 mg; Mn (MnSO4·5H2O), 45 mg; Zn (ZnO), 100 mg; Cu (CuSO4·5H2O), 20 mg; I (KI), 0.70 mg; Se (Na2SeO3·H2O), 0.25 mg; vitamin A, 10,000 IU; vitamin D3, 2,500 IU; vitamin E, 100 IU; vitamin K, 10IU; vitamin B2, 10 mg; vitamin B6, 1 mg; vitamin B12 50ug; biotin, 80ug; folic acid, 5 mg; nicotinic acid, 15 mg; choline chloride 1500 mg.

### Reproductive performance

After farrowing, the total number of newborn piglets/litter, stillbirth piglets/litter, and litter sizes at weaning were recorded. In addition, the average piglet birth weight/litter before suckling, and on d 14 and 21 (weaning) were recorded, and average daily gain of piglets/litter during d 0–14, 14–21, and 0–21 were recorded. Cross-fostering within diet groups was conducted within 24 h of birth to ensure even numbers of piglets per sow.

### Sample collection

A 5 mL blood sample was collected from the marginal ear veins of each sow at farrowing and on d 14 of lactation. Plasma samples were then obtained by centrifugation at 3,000 × *g* for 10 min at 4 °C and immediately stored at −80 °C for subsequent analysis.

Milk samples were manually collected from 4–6 mammary glands on d 7, 14, and 21 of lactation, following an intramuscular injection of oxytocin to induce milk let down. All the milk samples were stored at −80 °C until analysis. Average milk production per sow during d 0–14, 14–21, and 0–21 of lactation was calculated using the average daily gain per piglet multiplied by the litter size and constant “4”^38,39^.

### Plasma parameters

HDL-C, LDL-C, total CHO, and triglyceride in plasma were measured using an instrument (Biochemical Analytical Instrument, Beckman CX4, Beckman Coulter Inc., Brea, CA) and commercial kits (Sino-German Beijing Leadman Biotech Ltd., Beijing, China).

### Determination of lipid profiles in milk

The proportion of fat in milk was analysed using a MilkoScan FT120 infrared automatic analyzer (Foss, Hillerød, Denmark).

Extraction and lipid methylation in milk were performed in duplicate from 200 μL samples, according to the method of Lepage and Roy^40^, which recommends treatment with 2 mL methanol: toluene 4:1 (*v*/*v*) solution, and transmethylation with boron trifluoride (BF_3_) and methanolic KOH^41^. Fatty acids profiles were determined using gas chromatography (Agilent 6890, Boston, MA) equipped with a 100 m × 0.25 mm × 0.2 μm film-fused silica capillary column (SP1233, Supelco Inc., Bellefonte, PA, USA) and a flame ionisation detector. Injector and detector temperatures were 280 °C. The column temperature was maintained at 140 °C for 5 min, then increased at a rate of 3 °C/min to 220 °C and held for 40 min. Individual fatty acid peaks were identified by comparison with known reference methyl esters. All fatty acid values were expressed as a proportion of total fatty acids.

### Statistical analysis

All results are expressed as means and standard errors. Statistical analyses were conducted using SAS 8.2 (SAS Institute, Inc.). Normality of the data and homoscedasticity were checked using standard tests. All data were analysed using one-way analysis of variance (ANOVA), and feeding sequence was used as the independent variable. Differences were considered statistically significant at *P* < 0.05.

### Implications

Maternal two-meal feeding sequences with varying crude protein reduced the plasma cholesterol levels, increased milk production, and improved milk lipid profiles of sows, which contributed to the health of sows and played a key role in increasing piglet growth performance.
